# Fibrin Clot Augmented Meniscal Repair: A Technique to Enhance Healing After Bucket Handle Medial Meniscus Repair

**DOI:** 10.7759/cureus.107867

**Published:** 2026-04-28

**Authors:** Samir Ben Salah, Ayman Ben Abdellah, Achraf Tebbaa El Hassali, Najib Abdeljaouad, Hicham Yacoubi

**Affiliations:** 1 Department of Orthopedic Trauma, Mohammed VI University Hospital, Oujda, MAR; 2 Faculty of Medicine and Pharmacy, Mohammed First University, Oujda, MAR; 3 Department of Orthopedics and Traumatology, Mohammed VI University Hospital, Oujda, MAR

**Keywords:** augmented, bucket handle, fibrin clot, medial meniscus, meniscal repair

## Abstract

Meniscal repair in the avascular “white-white” zone remains challenging because of its limited intrinsic healing potential. We report our augmentation technique using a fibrin clot in association with meniscal suturing. This is a single case report of a 25-year-old high-level athlete presenting with a bucket-handle tear of the medial meniscus of the left knee. Arthroscopic repair was performed using an outside‑in suture technique, combined with the interposition of an autologous fibrin clot within the repair site. Postoperative management included early passive mobilization and non-weight-bearing for six weeks. Functional outcome was assessed using the Tegner-Lysholm score, which showed an excellent result, allowing the patient to return to pain-free sports activity one year after surgery. This case suggests that fibrin clot augmentation is a simple, low-cost, and potentially effective biological adjunct to enhance healing after meniscal repair, even in poorly vascularized zones.

## Introduction

Meniscal preservation has become a major objective in the management of knee injuries, especially in young and athletic patients [1]. Unlike partial or total meniscectomy, which is associated with an increased risk of early femorotibial osteoarthritis, meniscal repair aims to maintain the biomechanical role of the meniscus in load distribution, joint stability, and cartilage protection [1,2]. However, the healing potential of meniscal tears remains debated, particularly when the lesion is located in the avascular “white-white” zone, where the absence of blood supply is thought to limit tissue regeneration. To overcome this limitation, several biological augmentation techniques have been proposed, including intra-articular injection of blood derivatives or the local application of fibrin clot or platelet concentrates at the repair site [2,3]. In this context, we report a case of a young high-level athlete with a bucket-handle tear of the medial meniscus treated by inside-out repair augmented with a fibrin clot, and we describe the surgical technique and clinical outcome, highlighting the potential interest of this simple, low-cost method to enhance meniscal healing [2-4].

Overall, failure rates of meniscal repairs are often reported between 10% and 25%, with lower values in specialized centers or when modern techniques are used, and higher rates for isolated medial repairs, bucket-handle tears, and very long-term follow-up. Medial location, absence of anterior cruciate ligament reconstruction, older techniques, and a long delay before surgery increase the risk of failure [4,5].

Fibrin clot augmentation of meniscal repair is biologically logical and has been associated with good outcomes in many case series, especially for high-risk lesions. However, controlled trials are scarce, and systematic reviews consider the available evidence to be limited and heterogeneous. It can be stated that fibrin clot use may improve the healing potential, but this benefit has not yet been firmly demonstrated for all situations [5,6].

## Case presentation

A 25‑year‑old male competitive athlete, with no significant medical history, sustained a sports injury with an imprecise mechanism. He presented with a medial meniscal syndrome of the left knee, combining pain and episodes of intermittent locking. Clinical examination revealed tenderness over the medial joint line of the left knee and a positive grind test in internal rotation.

**Figure 1:**
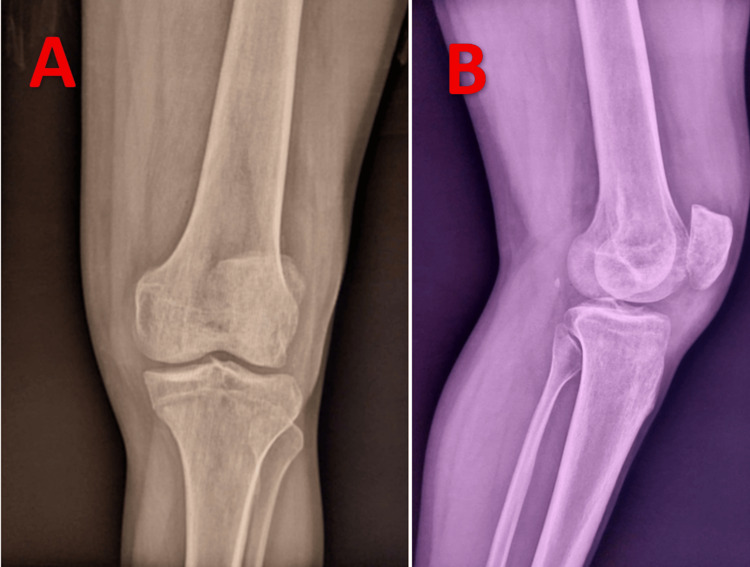
Standard anteroposterior (A) and lateral (B) radiographs showing no abnormalities.

**Figure 2:**
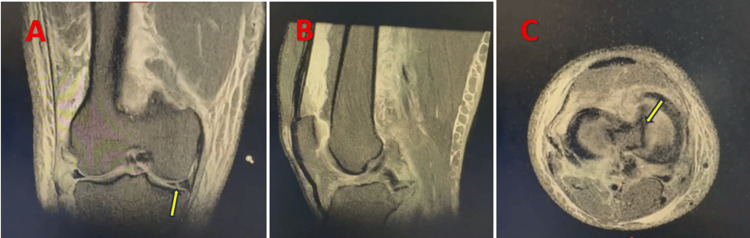
Radiological findings (MRI) confirming the meniscal lesion. (A) Coronal slice through the meniscus showing the lesion (yellow arrow) (B) Sagittal slice through the intercondylar notch (C) Axial slice through the meniscus demonstrating the displaced bucket-handle fragment (yellow arrow)

Radiological assessment included standard anteroposterior and lateral radiographs (Figure 1), as well as an MRI of the left knee, which confirmed a medial meniscal tear (Figure 2).

The surgical procedure was performed under spinal anesthesia, with the patient positioned supine on a standard table, using a flexion support and a second support against the lateral aspect of the knee to allow forced valgus, with a pneumatic tourniquet applied at the root of the thigh. We then carried out the antero-external and antero-internal approaches under arthroscopic control.

We began with joint exploration, which revealed presence of a bucket-handle tear of the medial meniscus luxated into the notch, which we easily reduced using a palpating hook, absence of chondral lesions, and intact central pivot.

**Figure 3:**
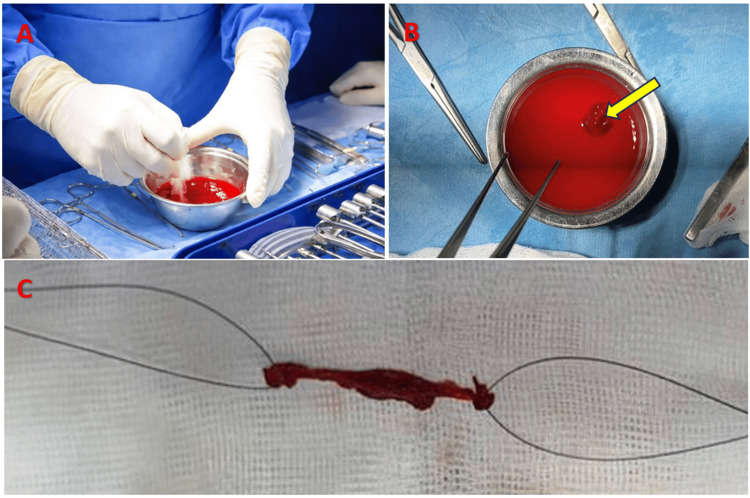
Intraoperative images showing the steps of clot preparation (A) Gentle manual centrifugation of the blood sample in normal saline (B) Isolation of the fibrin clot as a single mass (C) Placement of the clot on two sutures, one at each end

**Figure 4:**
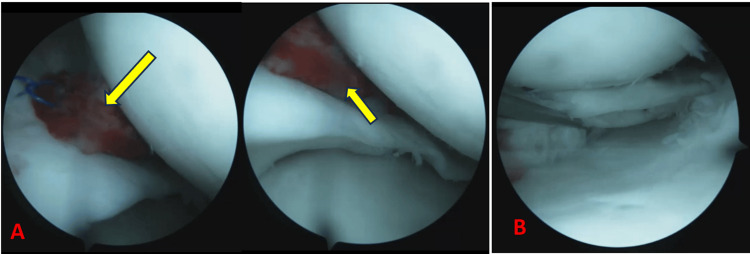
Intraoperative arthroscopic images (A) Placement of the fibrin clot into the bucket-handle tear (yellow arrows) (B) Arthroscopic view after meniscal suturing

In parallel with the exploration and meniscal preparation for suturing, one member of our team prepared the clot: (1) collection of 50 mL of the patient’s blood; (2) manual centrifugation for 10 minutes in a cupule with saline solution until clot formation, as documented in Figure 3; and (3) flattening of the clot and placement on two sutures, one at each end (Figure 4).

The clot was then introduced through the internal approach, positioned sagittally within the meniscal lesion, and fixation of both (meniscus and clot) was achieved with four stitches using the outside-in technique.

The procedure lasted 1 hour and 30 minutes, with a tourniquet time of one hour.

Postoperative rehabilitation consisted of early passive range‑of‑motion exercises initiated once pain had subsided, with non‑weight‑bearing maintained for six weeks.

Functional outcome was assessed using the Tegner-Lysholm score and was excellent. The patient was able to resume his sporting activities one year after surgery.

You will find here a video detailing all the steps of the procedure (Video 1).

**Video 1:** A video detailing all the steps of the procedure

## Discussion

The healing potential of meniscal tears, especially those located in the avascular “white-white” zone, remains controversial [1,2]. Traditionally, the absence of vascular supply in this region has led many authors to consider the chances of healing as limited, which long justified the widespread use of partial or total meniscectomy [2]. However, advances in our understanding of meniscal biomechanics have radically changed this perspective [2,3]. The meniscus is now recognized as an essential structure for load distribution, shock absorption, joint stability, and protection of the articular cartilage. In young and athletic patients, the removal of meniscal tissue is clearly associated with an increased risk of early knee osteoarthritis and functional limitations, which has favored a paradigm shift toward meniscal preservation whenever possible [3].

In this context, biological augmentation techniques have been developed to improve the healing environment of repaired menisci, particularly in poorly vascularized zones [3,4]. Among these techniques, intra‑articular injections of platelet‑rich plasma or fresh frozen plasma, and the use of fibrin clots placed directly at the site of the tear, have been described [4]. Fibrin clots provide a scaffold rich in fibrin and cellular elements that may promote cell migration, proliferation, and matrix synthesis at the repair site [4,5]. They also serve as a mechanical support integrated into the suture construct, potentially improving the stability and biological quality of the repair [5,6].

In our case, we used a fibrin clot incorporated into an inside‑out meniscal suture technique for a bucket‑handle tear in a young high‑level athlete. The procedure was simple, reproducible, and did not require expensive equipment. The postoperative course was uneventful, and with a standardized rehabilitation protocol, the patient achieved an excellent Tegner-Lysholm score and was able to return to sport without pain or functional limitation. This favorable outcome suggests that fibrin clot augmentation can be a valuable adjunct to meniscal repair, even in tears involving areas traditionally considered to have poor healing potential.

Nevertheless, our observation is limited to a single case, with inherent limitations in terms of generalizability. The promising functional result must be interpreted with caution. Larger prospective series and comparative studies are needed to confirm the real benefit of fibrin clot augmentation versus standard meniscal repair, to better define the indications (type of tear, location, age, and sport level), and to standardize the surgical and rehabilitation protocols. Despite these limitations, our case contributes to the growing body of evidence supporting biologically augmented meniscal preservation strategies and reinforces the idea that every effort should be made to save meniscal tissue in young and active patients [6].

## Conclusions

Meniscal repair, even for tears located in the white-white zone, is now increasingly preferred in order to preserve meniscal tissue and reduce the risk of knee osteoarthritis, particularly in young patients. Fibrin clot augmentation represents a simple, reproducible, and potentially effective option to promote healing. In our case, this technique allowed a return to sports with an excellent functional outcome, reinforcing the interest in such biologic methods to optimize meniscal repair.
